# Serum tryptase and IgE before and after explantation in women with breast implant illness

**DOI:** 10.1016/j.jpra.2026.07.036

**Published:** 2026-07-31

**Authors:** Siham Azahaf, Jinske Hoff, Karlinde A. Spit, Christel J.M. de Blok, Thomas Rustemeyer, Prabath W.B. Nanayakkara

**Affiliations:** a Section General Internal Medicine, Department of Internal Medicine, Amsterdam University Medical Centre, Amsterdam, the Netherlands; b Vrije Universiteit, Amsterdam, the Netherlands; c Department of Dermatology and Allergology, Amsterdam University Medical Centre, Amsterdam, the Netherlands

**Keywords:** Breast implant illness, Immunoglobulin E, Tryptase, Explantation, Mast cell activation, Chronic inflammation

## Abstract

**Background:**

Breast Implant Illness (BII) is characterized by systemic and local symptoms in a subset of women with silicone breast implants, but its pathophysiology remains unclear. Mast cell activation has been proposed as an underlying mechanism, yet systemic mast cell–related biomarkers have not been systematically evaluated in this context. This study aimed to assess whether explantation is associated with changes in serum tryptase and total IgE in women with BII.

**Methods:**

Seventy-one women with BII at a tertiary outpatient clinic provided paired blood samples before and after explantation (median interval, 8 months). Serum tryptase was measured by enzyme-linked fluorescence assay and total immunoglobulin E (IgE) by ImmunoCAP/EliA. Paired changes were analyzed in the overall cohort and in baseline-defined subgroups, including allergy status and pre-explantation IgE >100 kU/L.

**Results:**

In the full cohort, neither serum tryptase nor total IgE changed significantly after explantation (median differences: +0.09 ng/mL and –1.2 kU/L, respectively; both p > 0.1). Among 13 women with high baseline IgE (>100 kU/L), nine showed declines (median –118 kU/L), whereas tryptase levels remained unchanged and within normal limits. Of five women with elevated baseline tryptase (>11.4 ng/mL), three showed decreases after explantation, but only one normalized.

**Conclusions:**

These findings show no evidence of explantation-associated changes in serum tryptase in women with Breast Implant Illness. Total IgE levels showed no consistent change overall, although heterogeneous post-explantation IgE changes were observed in a small subgroup with elevated baseline IgE. Mast cell involvement in BII cannot be excluded, as local mast cell activity and other mast-cell–derived mediators were not assessed.

## Introduction

Breast Implant Illness (BII) describes systemic and local symptoms reported by a subset of women with silicone breast implants.^1^ Systemic symptoms include debilitating fatigue, night sweats and arthralgia, accompanied by local symptoms such as breast pruritus and axillary lymphadenopathy. Symptoms typically manifest more than ten years after implantation and may improve following implant removal (explantation).^2^, ^3^, ^4^ Yet, the underlying etiology remains unknown and no diagnostic biomarkers or validated criteria are available. This uncertainty complicates clinical decision-making regarding explantation.^5^

Several mechanisms have been proposed to explain the pathophysiology of BII, such as Autoimmune/Inflammatory Syndrome Induced by Adjuvants (ASIA), biofilm-induced chronic inflammation, and chronic inflammatory responses to silicone or other implant-derived compounds.^6^, ^7^, ^8^, ^9^, ^10^, ^11^

Nagy et al. recently hypothesized that symptoms attributed to BII may, in some women, reflect mast cell activation syndrome (MCAS) triggered by silicone breast implants.^12^ Foreign bodies have been reported to exacerbate MCAS, with implant removal often providing symptom relief.^13^ In support of this hypothesis, Nagy et al. demonstrated increased mast cell density in periprosthetic capsule tissue from women with BII compared with implant recipients without BII and controls, suggesting increased local mast cell presence and activation.^12^ However, this study did not formally assess MCAS, nor did it evaluate whether peri-implant mast cell activation translates into systemic mast cell mediator release. Notably, women with BII often report new or worsening allergies, raising the possibility of mast cell involvement.^14^

Formal diagnosis of MCAS relies on event-related biochemical evidence, including transient increases in serum mast cell mediators.^15^ Applying such criteria in women with BII is challenging, as symptoms are typically chronic rather than episodic and baseline or event-related tryptase measurements are often unavailable. Rather than aiming to diagnose MCAS, systemic mast cell involvement may therefore be explored using established biomarkers that reflect mast cell burden or activity over time.

Serum tryptase is the most specific and widely used biomarker of systemic mast cell burden.^16^ Serial assessment of serum tryptase may therefore provide insight into whether basal systemic mast cell–related signals change after explantation. Given that women with BII frequently report new or worsening allergic symptoms, total immunoglobulin E (IgE) was included to help interpret serum tryptase findings in relation to concomitant IgE-mediated allergic or atopic conditions, and to assess whether IgE levels change after explantation.

Against this background, this study aimed to assess whether explantation is associated with changes in serum tryptase and total IgE levels in women with BII.

## Method

We conducted an observational cohort study of women with BII who underwent explantation of silicone breast implants (SBI) between 2021 and 2024. Patients were evaluated at a specialized silicone outpatient clinic within the Department of Internal Medicine at Amsterdam University Medical Center. This clinic focuses on the evaluation and management of women with local and systemic symptoms attributed to silicone breast implants. A national guideline for the evaluation and management of systemic symptoms associated with silicone breast implants, developed by our group, is accessible online.^17^ Inclusion criteria were age >18 years, diagnosis of BII according to our protocol,^4^^,^^17^ and availability of paired pre- and post-explantation blood samples (> 6 months apart). Ethical approval was obtained (ref: 2024.0730), and all participants provided written informed consent.

Patients were routinely evaluated at the silicone outpatient clinic both before and after explantation. Symptom status was reassessed during post-explantation follow-up visits based on clinical consultation and documentation in the electronic medical record. For subgroup analyses, symptom improvement was defined as a clinically meaningful improvement in the majority of self-reported debilitating symptoms compared with baseline.

Serum tryptase and total immunoglobulin E (IgE) were measured before and after explantation. Tryptase was analyzed by enzyme-linked fluorescence assay (ELFA), and IgE by ImmunoCAP/EliA (Phadia250 analyser). Clinical and demographic data were extracted from electronic hospital records. Extracted variables included age, body mass index, smoking status, alcohol use, year of implantation, Pre- and post-explantation symptoms, duration of symptoms, timing of blood sample collection, comorbidities, allergies, antinuclear antibodies (ANA) status, reason for implantation, rupture history, silicone leakage, implant location, and implant brand when available.

### Statistical analysis

Normality of paired differences was tested with Shapiro–Wilk. Paired t-tests were used when normally distributed; otherwise, Wilcoxon signed-rank tests were applied. Subgroup analyses examined outcomes by baseline-defined characteristics, including allergy status and baseline IgE levels; analyses in subgroups defined by post-explantation symptom improvement were descriptive. Significance was set at p<0.05. Analyses were conducted in R (version 4.2.1; R Core Team, 2022).

## Results

Seventy-one women were included (Table 1), of whom 11 had implant-based reconstruction. Median implantation time was 13 years, and median symptom duration was 6 years. The most common implant brands were Allergan (41%), Eurosilicone (14%), and Mentor (13%). Symptom improvement after explantation was reported in 70%. Forty-eight women (68%) reported allergies (Appendix Table S1), with 23 noting worsening after implantation; 15 of these (65%) improved following explantation. The median interval between blood samples was 12 months (IQR 3), with a median of 8 months (IQR 2; range 6–13) between explantation and the second sample.

**Table 1:** Baseline characteristics (n=71).

**Age, mean, MAD**	46, 7
**BMI, median, IQR**	24, 5
**Smoking, n (%)**	
Never	37 (52)
Current	9 (13)
Former	25 (35)
**Pack years, median, IQR**	10, 10
**Alcohol**	
Yes	42 (59)
No	29 (41)
**Units per week (n=42), median, IQR**	1, 4
**Years of Implantation, median, IQR**	13, 10
**Years of complaints, median, IQR**	6, 7
**Improvement after explantation, n (%)**	
Yes	50 (70)
No	21 (30)
**Median time between 1^st^ and 2^nd^ blood collection, IQR**	12, 4
**Median time between explantation and 2^nd^ blood collection, IQR**	8, 2
**Allergies, n (%)**	
Yes	48 (68)
No	23 (32)
**Allergy categories, n** (**%**)†	
Metals	25 (24)
Airborne allergies	23 (22)
Pharmaceutical agents	19 (18)
Food-related allergies	10 (9)
Preservatives / Chemicals	4 (3)
Other	25 (24)
**Worsening of allergies during implantation, n (%)**	
Yes	23 (48)
No	25 (52)
**Improvement of allergies after explantation, n (%)**	
Yes	15 (65)
No	8 (35)
**Atopy, n (%)**	
Yes	28 (39)
No	43 (61)
**ANA positive, n (%)**	13 (18)
**Reason for implantation, n (%)**	
Reconstructive	11 (15)
Cosmetic	60 (85)
**Rupture history, n (%)**	15 (21)
**Silicone leakage, n (%)**	22 (31)
**Location of implants, n (%)**	
Submammary	17 (24)
Subpectoral	43 (61)
Dualplane	7 (10)
Unknown	4 (6)
**Implant brand, n (%)**	
Allergan	29 (41)
Eurosilicone	10 (14)
Mentor	9 (13)
Cereform	1 (1)
PIP	1 (1)
Arion	1 (1)
Sebbin	2 (3)
CUI	2 (3)
Unknown	16 (23)
**Bilateral, n (%)**	67 (94)
**Unilateral, n (%)**	4 (6)
**Autoimmune disease, n (%)**‡	5 (7)
**Symptoms before explantation, n (%)**	
Fatigue	68 (96)
Local complaints	61 (86)
Sicca complaints	52 (73)
Myalgia	49 (69)
Joint pain	43 (61)
Cognitive impairment	43 (61)
Morning stiffness	40 (56)
Dyspnea	36 (51)
Pruritus§	33 (47)
Night sweats	33 (47)
Rashes¶	33 (47)
Hair loss	33 (47)
Weight change	27 (38)
Sleeping disturbances	20 (28)
Flu like symptoms	9 (13)
Capsular contracture (Baker 3 or 4)	13 (19)
Palpitations	13 (19)
Palpable (painful) lymph nodes	1 (1)

† Some patients had >1 allergies (total n=106), see appendix Table 1 for overview.

‡ M. Sjogren, Lupus profundus/tumidus, Diabetes type 1, Rheumatoid arthritis, Hashimoto, Inflammatory Bowel Disease

§ 24 women (73%) had an allergy, 9 women (27%) did not.

¶ 23 women (70%) had an allergy, 10 women (30%) did not.

The most frequently reported pre-explantation symptoms were fatigue (96%), local complaints (86%), sicca complaints (73%), myalgia (69%), joint pain (61%), and cognitive impairment (61%). Silicone leakage, identified by MRI, ultrasound, or intraoperative confirmation of implant rupture during explantation, was observed in 31% of patients. Brands included Natrelle/Allergan/McGhan/Inamed (n=9), Mentor (n=2), Eurosilicone (n=2), Arion (n=1), Cereform (n=1), Sebbin (n=1), CUI (n=1), and unknown brand (n=5).

### Tryptase levels

In the full cohort, the median paired difference in serum tryptase was +0.09 ng/mL (IQR 0.67; Wilcoxon signed-rank p = 0.18). Among women with reported allergies, the median paired difference was +0.18 ng/mL (IQR 0.83; p = 0.16). In women reporting symptom improvement and in those reporting improvement of allergy-related symptoms, median paired differences were +0.22 ng/mL (IQR 0.71) and +0.28 ng/mL (IQR 0.88) (Table 2).

**Table 2:** Tryptase levels (ng/mL) in women with BII pre- and post-explantation.

**Group**	**n**	**Pre median** (**IQR**)†	**Post median (IQR)**	**Median Δ (IQR)**	**p-value**
Full cohort	71	5.0 (3)	5.4 (3)	+0.09 (1)	0.18
Symptom-improvers	50	4.9 (3)	5.1 (3)	+0.22 (1)	n.a
Allergies	48	5.5 (2)	5.5 (3)	+0.18 (1)	0.16
Allergy-improvers	15	5.8 (3)	5.7 (3)	+0.28 (1)	n.a

† IQR’s rounded off to nearest whole numbers, tryptase reference values: 0 - 11.4 ng/mL, n.a.= not applicable

We identified five women (2 reconstruction) with elevated pre-explantation tryptase levels (>11.4 ng/mL). They all reported allergies. Three women reported worsening of allergy related symptoms that improved after explantation. The other two women did not report worsening of allergy related symptoms. Serum tryptase declined in three patients (median 2.7 ng/mL, IQR 1.4), however only one fell below the normal upper limit; the other four remained above 11.4 ng/mL. Four out of five reported improvement of BII symptoms after explantation, including the women with allergy symptom improvement.

### IgE Levels

Total IgE showed a paired median change of −1.2 kU/L (IQR 14; p = 0.16) in the overall cohort (n = 71). Among women with reported allergies, the median paired change was −1.8 kU/L (IQR 18; p = 0.22). In women reporting symptom improvement and in those reporting improvement of allergy-related symptoms after explantation, median paired changes were −1.3 kU/L (IQR 17) and −5.9 kU/L (IQR 17), respectively (Table 3).

**Table 3:** IgE levels (kU/L) in women with BII pre- and post-explantation.

**Group**	**n**	**Pre median** (**IQR**)†	**Post median (IQR)**	**Median Δ (IQR)**	**p-value**
Full cohort	71	38.6 (52)	35.3 (62)	-1.2 (14)	0.16
Symptom-improvers	50	39.7 (69)	35.3 (68)	-1.3 (17)	n.a.
Allergies	48	45.1 (45)	40.5 (57)	-1.8 (18)	0.22
Allergy-improvers	15	58.3 (188)	55.2 (89)	-5.9 (17)	n.a.

† IQR’s rounded off to nearest whole numbers, IgE reference values: 0 - 100 kU/L, n.a.= not applicable

Women with reported allergies had significantly higher baseline IgE than non-allergic women (median 45.1 vs. 31.8 kU/L; Wilcoxon signed-rank p = 0.04), however neither the within-group median IgE changes pre- and post explantation (–1.8 kU/L vs. +0.1 kU/L) nor the between-group difference in these changes reached statistical significance (all p > 0.1).

In 13 women (1 reconstruction) with pre-explantation IgE >100 kU/L, nine exhibited a post-explantation decrease in IgE and four an increase. Among the nine decliners, the mean paired difference in IgE was –125 kU/L. Of these nine women, three had no documented allergies, three reported worsening of allergy related symptoms which improved in two patients after explantation. Eight women reported improvement of BII symptoms.

In the same subgroup, none of the women had serum tryptase levels >11.4 ng/mL. Tryptase levels showed no meaningful change after explantation (median pre-explantation 3.54 ng/mL (IQR 1.97) vs. post-explantation 4.01 ng/mL (IQR 1.51); median paired difference 0.05 ng/mL (IQR 0.70); p = 0.64). Tryptase levels decreased in four women.

## Discussion

In this study, we compared paired serum tryptase and total IgE levels before and after explantation in 71 women with Breast Implant Illness (BII). No significant changes were observed in the overall cohort or in baseline-defined subgroups, and no consistent differences were observed in women reporting symptom improvement. Among the 13 women with high pre-explantation IgE (>100 kU/L), nine showed a decline after explantation, while tryptase levels remained unchanged.

Although Nagy et al.^12^ demonstrated increased mast cell density in periprosthetic capsule tissue of women with breast implants, our findings do not support a role for systemic mast cell activation, as assessed by serial serum tryptase measurements, in the pathophysiology of BII. Most serum tryptase values remained below the upper limit of normal (<11.4 ng/mL) both before and after explantation, and no consistent declines were observed among women reporting symptom improvement.¹¹ While serum tryptase may remain within the normal range in mast cell activation, the absence of consistent within-person changes following explantation argues against a clinically relevant systemic mast cell signal associated with implant presence. Nevertheless, mast cell involvement in BII cannot be completely refuted, as mast cells were not assessed directly in tissue and other mast-cell mediators were not measured.

Mast cells have a prominent role in the process of wound healing and are found in increased numbers at the site wound healing responses.^18^ A strong association has been found between fibrin and mast cell activation suggestive of a prominent role in the fibrotic response and thus capsular formation of biomaterials.^19^ Mast cell-deficient mice implanted with poly-L glycolic acid polymer showed to have thinner fibrotic capsules, lower cell density and lower collagen I, when compared to wild type mice.^20^ This suggests that mast cells not only stimulate inflammation but also alter the degree of fibrosis. Therefore it is possible that Nagy et al’s findings reflected this highly dynamical process. Mast cells play a multifaceted role in both the initiation and perpetuation of chronic inflammation. When activated, they release a variety of mediators that can initiate and modulate inflammatory responses. These mediators can cause inflammation, pain, and contribute to tissue damage.^21^ Local complaints such as capsular contracture are common among women with Breast Implant Illness, which may explain why the authors found higher levels of mast cells, reflecting local inflammation and/or excessive capsular formation.

The observation that some women with elevated IgE and allergy histories experienced declines after explantation warrants further study.^22^ Although IgE levels are subject to background biological variability and seasonal influences, we reviewed the timing of blood withdrawal and found no consistent seasonal pattern, making it unlikely that the observed changes can be fully attributed to seasonality alone. These findings suggest a temporal association between implant removal and changes in systemic IgE levels in a subset of patients. Reports of increased IgE levels in sera of 15 male and female burn patients with silicone tissue expanders for burn scars compared to controls without silicone tissue expanders (205.3 ± 56.9 vs. 32.4 ± 8.7 IU/ml; *p* < 0.001), suggest that foreign body exposure may influence systemic IgE levels, though mechanisms remain speculative.^23^

This study has limitations, including a modest sample size, two measurement time points, and non-standardized blood collection. In addition, not all self-reported allergies were confirmed by objective testing. Nevertheless, the paired pre- and post-explantation design in a well-characterized cohort provided a unique opportunity to directly evaluate the hypothesis of (explantation-responsive) systemic mast cell activation in BII using serum tryptase. However, other mast-cell mediators or (localized) mast-cell activity were not assessed and should be addressed in future studies.

In conclusion, we found no evidence of a systemic mast cell signal associated with implant presence in women with BII, as assessed by serial serum tryptase measurements. However, mast cell involvement in BII cannot be refuted, as mast cells were not assessed directly and other mast-cell–derived mediators were not measured; further investigation is warranted.

## Contributions

S.A.: conceptualization, study design, data collection, data analysis, data interpretation, writing—original draft, and final approval of the manuscript. J.H: data collection, writing—original draft, and final approval of the manuscript K.A. S.: writing—review and editing, and final approval of the manuscript. C.J.M.d.B.: writing—review and editing, and final approval of the manuscript. T.R: data interpretation, writing—review and editing. P.W.B.N.: data interpretation, writing—review and editing, supervision, and final approval of the manuscript.

## Ethical approval

Ethical approval was obtained (ref: 2024.0730), and all participants provided written informed consent.

## Funding

This research was financially supported by the silicone breast implant research program, coordinated by the National Institute of Public Health and the Environment on behalf of the Ministry of Health, Welfare, and Sport.

## Declaration of competing interest

The authors declare no conflict of interest in relation to this work.

## Appendix Supplementary materials

Image, application 1
