# Supplementary material for: Serum tryptase and IgE before and after explantation in women with breast implant illness
Source: JPRAS Open. 2026 Jul 31;51:723–30. doi: 10.1016/j.jpra.2026.07.036 (PMC13508643; doi:10.1016/j.jpra.2026.07.036)
Supplement: Supplementary file 1 [file mmc1.docx]

| **Table S1 Reported Allergens and Frequencies in Women With Breast Implant Illness** | | |
| --- | --- | --- |
| **Allergen** | **Frequency** | **Category** |
| house dust mite | 7 | Airborne allergens / Hay fever |
| hay fever | 13 | Airborne allergens / Hay fever |
| Christmas tree | 1 | Airborne allergens / Hay fever |
| allergic asthma | 2 | Airborne allergens / Hay fever |
| cats/dogs | 6 | Animal-related |
| plaster | 10 | Contact materials (skin) |
| latex | 3 | Contact materials (skin) |
| hair dye | 1 | Contact materials (skin) |
| mascara | 1 | Contact materials (skin) |
| detergent | 1 | Contact materials (skin) |
| shrimp | 1 | Food-related allergens |
| pine nuts | 1 | Food-related allergens |
| pork | 1 | Food-related allergens |
| peanut | 1 | Food-related allergens |
| banana | 1 | Food-related allergens |
| walnuts | 1 | Food-related allergens |
| wheat allergy | 1 | Food-related allergens |
| chocolate | 1 | Food-related allergens |
| tomato | 1 | Food-related allergens |
| bell pepper | 1 | Food-related allergens |
| penicillin | 3 | Medications |
| amoxicillin | 2 | Medications |
| antibiotics (not specified) | 4 | Medications |
| quinolones | 1 | Medications |
| morphine | 2 | Medications |
| NSAID | 6 | Medications |
| sulfasalazine | 1 | Medications |
| nickel | 17 | Metals |
| metals | 2 | Metals |
| amalgam | 1 | Metals |
| phenylmercury(II) acetate | 1 | Metals |
| titanium | 1 | Metals |
| chromium | 1 | Metals |
| iron | 1 | Metals |
| chromium | 1 | Metals |
| iodine | 2 | Other |
| methylisothiazolinone | 1 | Preservatives / chemicals |
| kathon | 1 | Preservatives / chemicals |
| thiomersal | 1 | Preservatives / chemicals |
| benzophenone 4 | 1 | Preservatives / chemicals |
| **TOTAL** | 106 |  |
